# Long-Term Cardiovascular Outcome in Children with MIS-C Linked to SARS-CoV-2 Infection—An Italian Multicenter Experience

**DOI:** 10.3390/biology11101474

**Published:** 2022-10-08

**Authors:** Nicoletta Cantarutti, Virginia Battista, Nicola Stagnaro, Marianna Eleonora Labate, Marianna Cicenia, Marta Campisi, Valerio Vitali, Aurelio Secinaro, Andrea Campana, Gianluca Trocchio, Fabrizio Drago

**Affiliations:** 1 Cardiology and Arrhythmias Complex Unit, Department of Paediatric Cardiology and Cardiac Surgery, Bambino Gesù Children’s Hospital, IRCCS, 00163 Rome, Italy; 2 Radiology Unit, IRCCS Istituto Giannina Gaslini, 16132 Genoa, Italy; 3 Department of Internal Medicine and Medical Specialities (DIMI), Clinic of Cardiovascular Diseases, University of Genoa, 16132 Genoa, Italy; 4 Advanced Cardiothoracic Imaging Unit, Department of Imaging, Bambino Gesù Children’s Hospital IRCSS, 00163 Rome, Italy; 5 Academic Department of Pediatrics, Bambino Gesù Children’s Hospital, IRCCS, 00163 Rome, Italy; 6 Cardiology Unit, IRCCS Istituto Giannina Gaslini, 16132 Genoa, Italy

**Keywords:** MIS-C, SARS-CoV-2, cardiovascular outcome, children, pediatric

## Abstract

**Simple Summary:**

MIS-C is a multisystem inflammatory syndrome that is characterized by severe systemic signs of inflammation and multi-organ failure, including the involvement of the heart. Our study described the long-term cardiovascular outcome in a cohort of pediatric patients with MIS-C, who were admitted to two Italian Pediatric Referral Centers. The number of pediatric patients with an MIS-C diagnosis was 67. Among them, 65% of them had ventricular dysfunction, 66% of them had pericarditis, 35% of them had coronary involvement, and 9% of them showed arrhythmias. Our data described many cases of myocarditis and pericarditis, with mild coronary involvement and a few cases of arrhythmias. This experience showed that cardiac involvement in MIS-C patients is almost the rule, and that LV dysfunction and pericarditis are the most frequent manifestations of it. However, the patients’ clinical course was satisfactory, and during the controls, no additional events or sequelae were observed apart from long-term myocardial scars in 50% of the patients that underwent CMR. Early treatment not only provides a prompt full recovery, but it also has a probably “protective” effect towards late adverse events and long-term complications.

**Abstract:**

MIS-C is a multisystem inflammatory syndrome that is characterized by multi-organ failure and cardiac involvement. The aim of this study was to describe the long-term cardiovascular outcome in a cohort of MIS-C pediatric patients, who were admitted to two Italian Pediatric Referral Centers. Sixty-seven patients (mean age 8.7 ± 4.7 years, male 60%) were included; 65 (97%) of them showed cardiac involvement. All of the patients completed one month of the follow-up, and 47% completed 1 year of it. ECG abnormalities were present in 65% of them, arrhythmias were present in 9% of them during an acute phase and it disappeared at the point of discharge or later. Pericarditis were detected in 66% of them and disappeared after 6 months. Coronaritis was observed in 35% of the children during an acute phase, and there were no more instances at the 1-year point. An LV dysfunction was present in 65% of the patients at the beginning of the study, with them having a full recovery at the point of discharge and thereafter. Elevated values of the NTproBNP and hsTp were initially detected, which progressively decreased and normalized at the points of discharge and FU. The CMR at the point of FU, there was a presence of long-term myocardial scars in 50% of the patients that were tested. No deaths that were caused by MIS-C during the FU were recorded. Cardiac involvement in MIS-C patients is almost the rule, but the patients’ clinical course was satisfactory, and no additional events or sequelae were observed apart from there being long-term myocardial scars in 50% of the patients that underwent CMR.

## 1. Introduction

MIS-C is the acronym for the Multisystem Inflammatory Syndrome in Children that is linked to the SARS-CoV-2 infection, and it is characterized by a severe systemic hyper-inflammation and multi-organ failure [1].

Cardiac involvement has already been described in patients with MIS-C; some reports have described children with severe ventricular dysfunction or cardiogenic shock, which have required mechanical ventilation, inotropic support or ECMO [2,3]. However, the etiopathogenesis of the ventricular dysfunction is not fully understood, and different pathophysiological mechanisms may be involved in this. An immune-mediate response that is triggered by the virus or a systemic inflammatory response may be responsible for this. The management of this condition is based on the expert consensus, and this includes the use of immunomodulatory agents, anticoagulation drugs and cardiac support [4].

The aim of this study is to describe the long-term cardiac involvement in a large cohort of pediatric patients with MIS-C who were admitted to two Italian Pediatric COVID-19 Referral Centers.

## 2. Materials and Methods

This is a multicenter Italian retrospective study, which included entirely consecutive symptomatic pediatric patients with an MIS-C diagnosis, who were admitted to Bambino the Gesù Children’s Hospital IRCCS and Giannina Gaslini Institute COVID Centers from March 2020 to May 2022. These “Referral Centers” were created for the SARS-CoV-2 pandemic in the Lazio and Liguria regions in March 2020. The clinical records were reviewed in order to identify all of the patients with an MIS-C diagnosis. 

The patients’ data, including their demographic information, contact history, previous history, clinical symptoms, medical treatment, imaging and laboratory findings during the hospital stay and follow-up (FU), were recorded. 

The laboratory tests for to detect SARS-CoV-2 were performed in accordance with the guidelines of laboratories that are identified by the Italian Health Ministry. Microbiological data accounted for the results of the nasopharyngeal, stool, urine, bone marrow and conjunctival swabs for the SARS-CoV-2 detection by the mean Real-Time Polymerase Chain Reaction (RT-PCR) and the antibodies for SARS-CoV-2 detection. The definition of MIS-C included the presence of a fever, laboratory evidence of inflammation and multisystem organ involvement without alternative plausible diagnoses, as well as evidence of a COVID-19 infection or recent exposure to a COVID-19 case. The diagnosis of MIS-C was confirmed based on the WHO criteria [1].

All of the patients were evaluated by a laboratory blood test, including by performing a multi-organ function assessment, and by observing the infection rates and inflammatory markers and cardiac biomarkers (NT-proBNP and troponin). Instrumental investigations such as an ECG, a chest X-ray and echocardiography were performed. The patients with cardiac involvement underwent continuous electrocardiographic monitoring and were daily revaluated with an ECG and echocardiography during the acute phase. Troponin was detected every 6 h if the level of it was elevated, and the NT-proBNP level was checked once a day.

In the patients with cardiac conduction or repolarization disturbances and/or supraventricular or ventricular hyperkinetic arrhythmias, 24 h ECG monitoring was performed.

Cardiac examinations, ECGs, echocardiography, 24 h ECG monitoring and cardiac biomarkers were performed before they were discharged and outside of the acute event.

The patients with acute cardiac involvement were regularly followed up at 1 month, 3 months, 6 months and until one year after the diagnosis with the following cardiac examinations: an ECG, a 2D Doppler echocardiogram and cardiac biomarkers. Cardiac magnetic resonance (CMR) was performed in the patients that were older than 8 years, within 2 weeks from the point of diagnosis, and after almost 6 months after the FU.

The statistical analysis was performed using SPSS Statistics 21 (IBM Corporation, Armonk, NY, USA). The categorical variables are expressed as absolute numbers or percentages. Continuous variables are reported as mean value ± standard deviation or median value ± interquartile range.

This study has been approved by the institutional committees of Bambino Gesù Children’s Hospital IRCCS (BGCH) and Giannina Gaslini Institute IRCCS (GGI) within the Research of the Ministry of Health (RCR-2021-23671212). The written informed consent was signed by the parents or legal guardians of the patients for invasive procedures or routine investigations to be performed that are not included in the routine management of all of the diseases. All of the investigations were conducted according to principles that are expressed in the Declaration of Helsinki.

## 3. Results

Between March 2020 and May 2022, 67 pediatric patients (mean age 8.7 ± 4.7 years, male 60%) with an MIS-C diagnosis were included in the current study, 65 (97%) of whom showed a cardiac involvement. The mean time that elapsed from an acute SARS-CoV-2 infection and an MIS-C presentation was 27 +/11 days. The mean FU was 9 ± 6.2 months. All of the patients completed one month of the FU, 87% of them completed 3 months of the FU, 58% of them completed 6 months of it, and 47% of them completed 1 year of the FU (Table 1). Not all of the children reached the 1-year FU point because more than half of the patients were enrolled in the last year.

**Figure 1:**
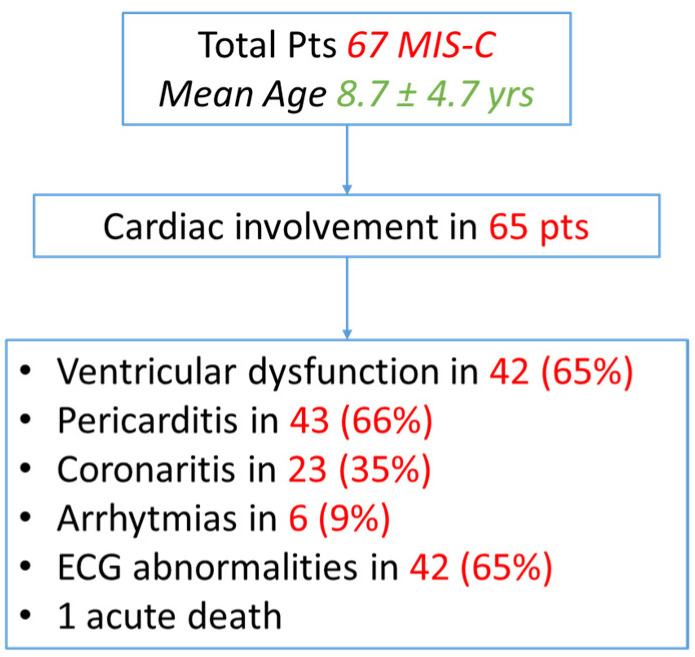
Flow chart of cardiac involvement in MIS-C patients.

Among them, during the acute event, 42 patients (65%) had ventricular dysfunction, 43 (66%) of them presented with pericarditis, 23 patients (35%) had a coronary involvement, and ECG abnormalities were observed in 42 patients (65%), and six children (9%) showed arrhythmias. One patient died of an acute respiratory failure and cardiogenic shock in the first 24 h of their hospitalization (Figure 1).

Pericarditis is defined as pericardial hyperechogenicity, and effusion was detected in 64% of the MIS-C patients, but after the treatment, it was completely resolved in all but 8% of the patients. During the FU, 2% of the patients still showed pericardial involvement at the 1- and 3-month-marks. Pericarditis was no longer observed after 6 months of the FU. In our cohort, no cases of severe pericardial effusion or tamponade that required pericardiocentesis were described.

Left ventricular (LV) dysfunction was present in 63% of the patients with MIS-C during the acute event. The patients with a reduced level of function showed a mean ejection fraction (EF) that was calculated to be 49 ± 14%.

A prompt LV function recovery was observed after corticosteroids and inotropes were administered; the mean EF at the point of discharge was 59 ± 9%, and only 3% of the patients showed mild LV dysfunction at the 1- and 3-month-marks of the FU. However, a complete normalization of the ventricular function was observed in all of the patients after 3 months of the FU, with a mean EF of 61 ± 3% at the 1-year-mark of the FU (Table 2 and Table 3).

Coronaritis, which is defined as a coronary inflammation, hyperechogenicity or dilatation, was detected in 34% of the children with MIS-C during the acute event. Coronary dilatations were diagnosed when the coronary arteries enlargement which was observed during the echocardiography was more than two standard deviations greater than the predicted mean (z-score >2). 

The coronary involvement included a coronary hyperechogenicity or a mild enlargement (no more than three of the z-score). No cases of a giant coronary aneurism were observed. At the point of discharge, 11% of the patients still showed a coronary involvement that persisted in 10% of them at the 1-month-mark, in 3% of them after 3 months, and in 2% of them after 6 months of the FU. Coronaritis was no longer detected at the 1-year-mark of the FU (Table 2).

At the point of onset, ECG abnormalities were observed in 64% of the patients with MIS-C, including ventricular repolarization anomalies and prolonged QTc intervals, which progressively decreased to 15% at the point of discharge. At the 1-month-mark of the FU only 3% of the patients showed anomalies, while no more ECG anomalies were detected at the following visits. 

Arrhythmias were present in 9% of the patients during the acute event, including non-sustained ventricular tachycardia (nsVT), supraventricular tachycardia (SVT) and premature ventricular beats (PVBs). All of the arrhythmias disappeared during the hospital stay, and they did not relapse during the FU after they were discharged.

Moreover, at the point of admission, the value of the NTproBNP was consistently high in the great majority of the patients (mean value: 9594 ± 10,542 pg/mL; median value: 4460 ± 1466 pg/mL). However, after the targeted therapy, the NTproBNP remarkably decreased (mean value at the point of discharge: 123 ± 143 pg/mL) and normalized as the FU progressed (mean values: 36 ± 28 pg/mL at 1 month, 53 ± 34 pg/mL at 3 months, 41 ± 22 pg/mL at 6 months and 35 ± 30 pg/mL at 1 year). 

Similarly, during the acute event, the value of the high-sensitivity troponin (hsTp) was high (mean value: 125 ± 241 pg/mL). The HsTp level became normal after a targeted treatment was administered (mean value at the point of discharge: 4 ± 4 pg/mL), thus remaining stable during the FU (Table 3).

**Figure 2:**
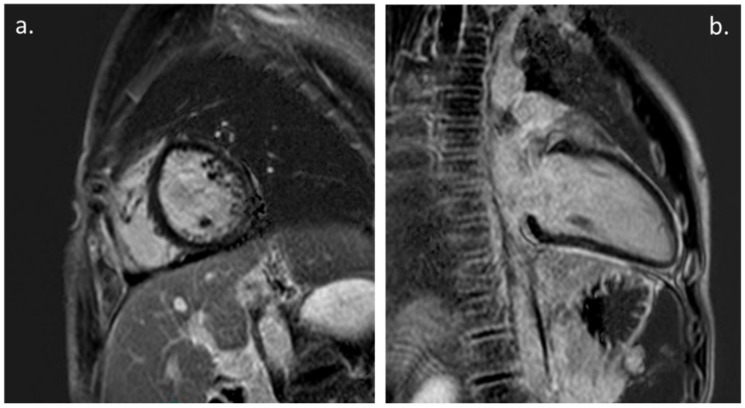
Cardiac Magnetic Resonance: (**a**). PSIR, short axis. Mild LGE with subepicardial pattern involving LV infero-lateral wall. (**b**). PSIR, LV 2-chamber. Mild intramyocardial LGE of the mid-basal inferior wall; also note, persistence of mild pericardial LGE.

The CMR was performed in 20 patients (31%) during the FU (Figure 2). The time between the onset of the symptoms and the FU CMR was 9.4 +/− 4.6 months. The CMR showed the presence of a myocardial LGE in 10 patients (15% of all of the patients and 50% of all of patients that underwent the CMR) and only one patient showed a persistent mild LV enlargement. No cases of LV dysfunction or myocardial edema were documented at the point of the CMR, and none of the patients showed evidence of a pericardial effusion.

All of the patients with MIS-C were treated with intravenous steroids (methylprednisolone 2 mg/kg i.v.) in association to intravenous immunoglobulin (Ig) 2 gr/kg and low-molecular-weight heparin (LMWH) 100 UI/kg s.c., and in most of the severe cases, with Anakinra 2 mg/kg s.c. The MIS-C patients with severe LV global systolic dysfunction (LV EF lower than 35%) and/or arrhythmias, hypotension or an overt MAS were simultaneously administered anakinra at 2 mg/kg/day, which was administered subcutaneously or, in the most severe patients, intravenously. Additionally, in the patients who did not improve within 24–48 h with the initially selected therapeutic intervention (Ig and corticosteroids), anakinra was administered [5].

In the case of a moderate-to-severe LV dysfunction we used inotropes, such as milrinone 0.5 mcg/kg/min i.v. and intravenous diuretics, such as furosemide. In cases of coronaritis, anti-aggregation therapy with acetylsalicylic acid (ASA) 3 mg/kg was prescribed. 

The patients were discharged and given a therapy with oral steroids, which were progressively tapered down and interrupted in 2–3 months. Only the patients with coronaritis and a Kawasaki-like disease were discharged also with an aspirin treatment that was suspended after the first 3 months of the FU.

All but one of the patients recovered after the therapy, and no deaths by SARS-CoV-2 were recorded during the FU. The only patient that died was a 4-year-old girl, with no previous disease history, who was transferred from another center 5 days after a respiratory failure event, who died within 24 h of their arrival for an acute respiratory failure and cardiogenic shock.

## 4. Discussion

In this study, we describe the long-term cardiac involvement in a cohort of pediatric patients with an MIS-C diagnosis who were admitted to two Italian Pediatric COVID-19 Referral Centers.

Cardiac involvement is frequently described in children with MIS-C, ranging from a myo-pericardial inflammation, a coronary dilation or an aneurysm to arrhythmias. The pathogenesis of MIS-C remains incompletely understood, but advances have been made in understanding the immune system components that are affected by the disorder and the immunomodulatory therapies which help patients to recover.

Cardiac support, immunomodulatory treatments, antiplatelet/anticoagulation drugs were used in the management of MIS-C in the acute phase [3]. The initial reports during the early phase of the SARS-CoV-2 pandemic documented that children were relatively spared from the severe manifestations of it but, more recently, severe cardiac involvement in children has been reported [6,7,8,9].

MIS-C is associated with high levels of inflammation, and it responds to anti-inflammatory therapies; it is therefore presumed to be immune-mediated. The patients with MIS-C variably demonstrated elevations that were above the normal range in multiple plasma cytokines and changes in the interferon responsiveness pathways. Moreover, there were demonstrate decreases in the monocyte and dendritic cell subsets, decreases in the absolute lymphocyte counts particularly with lymphopenia and an increased ability of spike-specific antibodies to activate monocytes in the cases of MIS-C [10].

LV systolic dysfunction is a feature that presented in many of the pediatric studies of MIS-C. the first case series reported a cardiac dysfunction in 75% of the patients [8]. More recent studies have showed different rates of LV dysfunctions in a different cohort of patients. In our previous multicenter pediatric study, an LV dysfunction was found in 59% of the patients with MIS-C [11]. Belhadjer et al. reported a selected cohort of 35 MIS-C patients who developed acute LV failure or shock, thus requiring mechanical ventilation and inotropic support or extracorporeal membrane oxygenation (ECMO) [2]. A high proportion of the patients had also been reported to have an elevated troponin level or BNP/pro-BNP values. Most of the patients experienced a complete full recovery of the ventricular dysfunction before they were discharged.

The mechanism an underlying myocardial dysfunction in MIS-C has not been yet fully elucidated. The different durations and presentations of a ventricular dysfunction may suggest that there are different pathophysiological mechanisms underlying it. In adults with a Sars-Cov2 infection, it has been reported that there were different causes of their myocardial injuries including an acute myocarditis, a hypoxic injury, an ischemic injury that was caused by cardiac microvascular damage, coronary artery disease, stress cardiomyopathy or systemic inflammatory response syndrome [7,12,13,14]. 

The first phase of an acute infection may be correlated to acute myocardial damage, whilst the second phase, which is characterized by a post-viral immunological reaction and systemic hyperinflammation, may be correlated to the development of myocardial inflammation and ventricular dysfunction [4,7].

In our case series, an LV dysfunction was present in almost 65% of the patients during the acute phase. No patients required an ECMO, however 32% of these children showed that they had moderate-to-severe dysfunction that required inotropic support. As reported in the literature [12], and also in our series, the elevated values of NTproBNP and hsTp correlated to the grade of the ventricular dysfunction. However, almost all of the patients showed a full recovery of the LV function before they were discharged.

The data about the frequency of pericardial manifestations are still unknown. The mechanism of pericarditis in COVID-19 is probably due to the inflammatory process of it and to the subsequent cytotoxic and immune-mediated effects that are related to a SARS-CoV-2 infection.

In the literature, one case of an acute pediatric pericarditis presenting with pericardial tamponade [15] and two cases of adolescents with an acute pericardial effusion were reported [16]. Our previous study showed that there was pericardial involvement in 50% of the children with a SARS-CoV-2 infection and in 66% of the patients with MIS-C [11].

Coronary artery dilation or aneurysms were described in around 6 to 30% of the patients [8,9,11,17]. Most of the case series described a mild and transient coronary artery dilation with detected z-scores of 2–2.5, suggesting that these findings may be related to coronary vasodilation as a compensatory phenomenon that is linked to the increased myocardial oxygen demand that is brought on by a fever and tachycardia, rather than by inflammation-related arterial wall damage. There were also some reports of large and giant coronary artery aneurysms [8,18] with the progression of them after the point of discharge.

In our experience, coronary involvement was observed in 34% of the patients during the acute event, including coronary hyperechogenicity or a mild enlargement (z-scores 2–3), while no cases of a worsening or giant aneurysm were described during the acute event and after 1 year during the FU. 

On the other hand, the most frequent findings were non-specific ECG anomalies and QTc prolongation [3]. Arrhythmic manifestations are reported in the literature in a wide range of percentages in around 7–60% of patients. First- and second-degree atrioventricular blocks were described in one study [18], while an atrial fibrillation was described in three reports [11,18,19]. There are also some studies reporting patients with non-sustained or sustained arrhythmias, who required, in some cases, ECMO support [3,8,18].

In our study, we found ventricular repolarization anomalies, and prolonged QTc, nsVT, SVT, and PVBs in 9% of the patients. All of these ECG anomalies disappeared before they were discharged and were not detected during the FU.

A previous CMR study reported a case series of four children and adolescents with COVID-19, showing that in the acute phase, the presence of a diffuse myocardial oedema with no evidence of an LGE was suggestive of replacement fibrosis or focal necrosis. These findings were correlated with post-infectious myocarditis [20]. On the contrary, in our study, the CMR documented the persistence an LGE that was indicative of myocardial scars in 50% of the patients that underwent the CMR at the 1-year-mark of the FU, without there being evidence of the symptoms, an LV dysfunction or arrhythmias. The meaning of these findings and their possible impact on a patient’s long-term outcome is actually unknown. We believe that this could be a reason to continue to conduct the follow-up of this subgroup of patients. 

The management of MIS-C patients requires a prompt approach. An inotropic support was used in the patients with moderate-to-severe ventricular dysfunction at the BGCH, together with diuretics, particularly in case of dysprotidemia [21]. At the GGI, no case of patients requiring inotropic therapy were described.

Many of the patients received an immunomodulatory therapy with intravenous immunoglobulin or corticosteroid [14,22]. Sometimes cytokine blockers were also used in addition to conventional therapy, particularly the IL-1 inhibitors (Anakinra) [18], particularly in the MIS-C patients with a severe LV global systolic dysfunction and/or arrhythmias and in patients who did not improve within 24–48 h after the initially selected therapeutic intervention was administered [5].

An antiplatelet treatment with aspirin was frequently used, particularly in the patients with evidence of coronary involvement or with a Kawasaki-like presentation [17,23]. A therapeutic or prophylactic anticoagulation with heparin was also used [14].

## 5. Conclusions

We can conclude that our experience showed that cardiac involvement in the MIS-C patients is unavoidable, and it is almost the rule. The LV dysfunction and pericarditis are the most frequent manifestations of this. However, the patients’ clinical courses were satisfactory, and no additional events were observed during the FU. The only long-term sequela was the presence of myocardial scars that were not associated with the LV dysfunction or arrhythmias in around 50% of the patients that underwent the CMR. The early treatment and identification of children that are at risk is mandatory for the patients’ recovery and the control of late adverse events. Further studies are needed to better define the management and long-term outcome of the patients with MIS-C, with particular regard to myocardial damage prognostic relevance.

## Figures and Tables

**Table 1:** MISC patients with cardiac involvement in follow-up.

Follow-Up Duration	MIS-C Patients (%)
1 month	65 (100%)
3 months	57 (87%)
6 months	38 (58%)
1 year	(47%)

**Table 2:** Cardiac involvement at onset and during follow-up.

	ACUTE PHASE	DISCHARGE	1 MONTH	3 MONTHS	6 MONTHS	1 YEAR
ECG anomalies	65%	15%	3%	0	0	0
Arrhythmias	9%	0	0	0	0	0
Pericarditis	66%	8%	2%	2%	0	0
Coronaritis	35%	11%	10%	3%	2%	0
LV Dysfunction	65%	3%	3%	0	0	0

Legend: ECG: electrocardiogram; LV: left ventricular.

**Table 3:** Myocardial involvement, therapy and outcome.

	ACUTE PHASE	DISCHARGE	1 MONTH	3 MONTHS	6 MONTHS	1 YEAR
Ejection fraction (mean)	49 ± 14 %	59 ± 9 %	61 ± 3 %	62 ± 4 %	62 ± 4 %	61 ± 3 %
NTproBNP (mean)	9594 ± 10,542	123 ± 143	36 ± 28	53 ± 34	41 ± 22	35 ± 30
hsTp (mean)	125 ± 241	4 ± 4	3± 4	3 ± 2	3 ± 2	3 ± 3
CMR LGE n * positive/n of CMR performed	0	0	0	0	0	10/20
Therapy	Inotropes Diuretics IVIG Aspirin LMWH Steroids Anakinra	Steroids Aspirin	Steroids Aspirin	Aspirin	No	no
Deaths	1	0	0	0	0	0

Legend: CMR: Cardiac magnetic resonance; hsTp: high-sensitivity troponin; LMWH: low-molecular-weight heparin; NTproBNP: B-type natriuretic peptide.

## Data Availability

The data presented in this study are available on request from the corresponding author. The data are not publicly available due to Institutional and Research policies.
